# Impact of Land Use on PM_2.5_ Pollution in a Representative City of Middle China

**DOI:** 10.3390/ijerph14050462

**Published:** 2017-04-26

**Authors:** Haiou Yang, Wenbo Chen, Zhaofeng Liang

**Affiliations:** 1 College of Forestry, Jiangxi Agricultural University, Nanchang 330045, China; yanghaiou2007@163.com; 2 Key Laboratory of Landscape and Environment, Jiangxi Agricultural University, Nanchang 330045, China; liangzhaofeng1989@163.com; 3 College of Tourism and Territorial Resources, Jiujiang University, Jiujiang 332005, China

**Keywords:** fine particulate matter (PM_2.5_), land use, land use regression (LUR), statistical analysis, urban functional zone

## Abstract

Fine particulate matter (PM_2.5_) pollution has become one of the greatest urban issues in China. Studies have shown that PM_2.5_ pollution is strongly related to the land use pattern at the micro-scale and optimizing the land use pattern has been suggested as an approach to mitigate PM_2.5_ pollution. However, there are only a few researches analyzing the effect of land use on PM_2.5_ pollution. This paper employed land use regression (LUR) models and statistical analysis to explore the effect of land use on PM_2.5_ pollution in urban areas. Nanchang city, China, was taken as the study area. The LUR models were used to simulate the spatial variations of PM_2.5_ concentrations. Analysis of variance and multiple comparisons were employed to study the PM_2.5_ concentration variances among five different types of urban functional zones. Multiple linear regression was applied to explore the PM_2.5_ concentration variances among the same type of urban functional zone. The results indicate that the dominant factor affecting PM_2.5_ pollution in the Nanchang urban area was the traffic conditions. Significant variances of PM_2.5_ concentrations among different urban functional zones throughout the year suggest that land use types generated a significant impact on PM_2.5_ concentrations and the impact did not change as the seasons changed. Land use intensity indexes including the building volume rate, building density, and green coverage rate presented an insignificant or counter-intuitive impact on PM_2.5_ concentrations when studied at the spatial scale of urban functional zones. Our study demonstrates that land use can greatly affect the PM_2.5_ levels. Additionally, the urban functional zone was an appropriate spatial scale to investigate the impact of land use type on PM_2.5_ pollution in urban areas.

## 1. Introduction

In recent years, the air pollution problem generated by unprecedented urbanization and economic growth in China has become one of the greatest urban issues, particularly fine particulate matter (PM_2.5_) pollution [1]. PM_2.5_, consisting of particles with aerodynamic diameters smaller than 2.5 μm, can absorb more hazardous substances than coarse particles and enter the human body by respiration, resulting in various respiratory and cardiovascular diseases [2]. Some epidemiological studies have confirmed that a long exposure to PM_2.5_ will greatly increase rates of cardiopulmonary morbidity and mortality [3,4]. Therefore, gaining a better and clearer understanding of PM_2.5_ pollution is of vital significance in preventing pollution and protecting public health. 

Numerous studies have been conducted on PM_2.5_, mainly focused on the spatial and temporal distribution [5,6,7,8,9,10], source apportionment [11,12,13,14], health effects [15,16,17,18], and estimation [19,20,21,22]. Studies have shown that at the macro-scale, PM_2.5_ pollution is significantly influenced by meteorological conditions [23,24,25,26]; at the micro-scale, PM_2.5_ pollution is strongly related to the land use pattern [27,28,29,30]. Some researchers have suggested that optimizing the land use pattern may mitigate PM_2.5_ pollution at a city or community level [31,32,33]. However, there are only a few researches analyzing the effect of land use on PM_2.5_ pollution and the consensus about the exact nature of their relationship has not yet been reached [28,34]. Thus, exploring the effect of land use on PM_2.5_ pollution seems to be urgent and significant.

To conduct research on the impact of land use on PM_2.5_ pollution, available PM_2.5_ data are critical. However, gaining access to enough PM_2.5_ data creates a big challenge. Several approaches have been developed over the last decade to solve this challenge, including spatial interpolation (e.g., kriging and inverse distance weighing), air dispersion models, and land use regression (LUR) models. The interpolation of pollutant concentrations is based on dense monitoring sites, while the routine monitoring sites are often too sparse. Dispersion models simulating the fate of pollution and transport can be useful, but are often infeasible at a high spatial resolution and are extremely dependent on accurate and spatially resolved input data [35,36]. In recent years, LUR models have been proved to be a valid and cost-effective alternative to these conventional approaches [37]. LUR models are statistical regression models based on a Geographical Information System (GIS) platform. They can be used to predict the concentration of atmospheric pollutants at a given site by establishing a statistical relationship between pollutant measurements and potential predictor variables, e.g., land use, traffic, and physical characteristics, etc. [37]. This approach was initially applied to air pollution in the SAVIAH (Small Area Variations In Air quality and Health) study [38]. Since then, it has gained an increasing amount of attention all over the world.

This paper therefore aims to employ LUR models and statistical analysis to explore the effect of land use on PM_2.5_ pollution in urban areas. Nanchang, the capital city of the Jiangxi province, was selected as a case study. It is a representative city of central China, but has been facing a serious PM_2.5_ pollution problem due to ongoing construction and heavy traffic. We applied LUR models to simulate the spatial variations of PM_2.5_ concentrations in the Nanchang urban area, analysis of variance and multiple comparisons to study the PM_2.5_ concentration variances among different types of urban functional zones, and multiple linear regression to investigate PM_2.5_ concentration variances among the same type of urban functional zones. The research results could help correctly understand the PM_2.5_ pollution pattern in urban areas. More importantly, they could provide a theoretical basis for urban PM_2.5_ pollution control.

## 2. Materials and Methods 

### 2.1. Study Area

**Figure 1:**
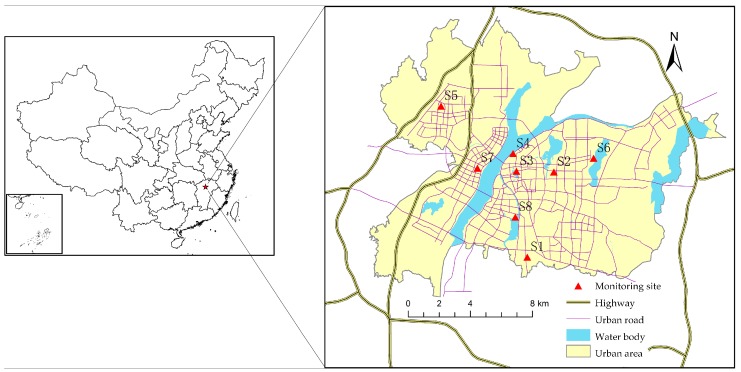
Map of the Nanchang urban area showing the monitoring site locations and road network.

Nanchang City (28°09′ N–29°11′ N, 115°27′ E–116°35′ E), the capital of the Jiangxi Province, China, is located in the southwest of Poyang Lake and the middle-and-lower reaches of the Yangtze River. It belongs to a subtropical monsoon climate zone, with an average annual temperature ranging from 17 to 17.7 °C and an annual precipitation value of 1600–1700 mm. Nanchang is an important transportation and shipping center in central China. Many highways and railways traverse this region. The city has experienced a rapid population growth and increase in vehicles in the past decade. By the end of year 2014, the residential population of Nanchang city was 5.24 million and the number of vehicles reached 618,100. All of these factors contribute to the tremendous flow of vehicles per day and the significant amount of pollutants such as PM_2.5_. The study was conducted in the Nanchang urban area that has been defined by the Land Use Planning, which covers an area of 562.46 km^2^. There are nine nation-standard PM_2.5_ monitoring sites defined by the China Environmental Monitoring Center (CEMC) reporting monitor data in the city on an hourly basis, and eight of them are located within the study area (Figure 1). The eight monitoring sites are located in different urban functional zones. S1 and S3 are located in the residential zones, S2 and S7 are in the educational zone, S4 and S6 are in the industrial zones, S8 is in the commercial zone, and S5 is in the control functional zone.

### 2.2. LUR Model Setting

The equation of the LUR models is expressed as follows:

(1) $$y=\beta_{0}+\beta_{1}X_{1}+\beta_{2}X_{2}+\ldots +\beta_{n}X_{n}+\varepsilon$$

where the dependent variable *y* is the pollutant concentrations, independent variables *X_1_...X_n_* are the potential variables, *β_1_...β_n_* are the associated coefficients, and *ε* is the constant intercept.

#### 2.2.1. Dependent Variable and Independent Variables

The monthly mean values of PM_2.5_ for the eight monitoring sites in 2014 were collected from the Nanchang Environmental Monitor Center (Table 1), and the specified monitoring site locations were also provided by the Monitor Center. 

The independent variables could be categorized into four classes: meteorological factors, traffic-related factors, land use factors, and population density. Circular buffers were created for 0.3, 0.6, 0.9, 1.2, 2.4, and 4.8 km radii using ArcGIS 10.2 (ESRI, Redlands, CA, USA). In total, 42 variables were used to build the LUR models. Each independent variable was explained as follows. A description of the independent variables is reported in Table 2.

Five meteorological variables were employed to characterize the weather conditions. They were relative humidity, air pressure, water vapor pressure, temperature, and wind speed. The monthly average values of the meteorological variables in 2014 were obtained from the Chinese Meteorological Data Share Service System (http://data.cma.cn/). 

The traffic-related variables included three subclasses: the intensity of main roads, intensity of secondary roads, and intensity of all roads. The road intensity was used to reflect the traffic conditions due to the unavailability of accurate traffic intensity data. Road intensity was computed by dividing the buffer area by the sum of road segments within the buffer. The data were collected from the transportation map of Nanchang urban master planning from 2011.

Three subclasses of variables including the ecological land proportion (green spaces, rivers, and lakes), industrial land proportion, and distance to large ecological space were used to describe the land use situation. The ecological land or industrial land in every buffer zone was calculated to obtain the values of the ecological land proportion or industrial land proportion. The straight-line distance of the monitoring site to the nearest large ecological space (Gan River, Qinshan Lake, Huangjia Lake, Yao Lake, Xiang Lake, Qian Lake, Aixi Lake, Diezi Lake, and Meiling Forest) was measured to describe the distance to a large ecological space. The data were derived from the Nanchang land use map of 2014 and satellite images from 2014. 

The residential land proportion was used to describe the population density as the population density was only available at a district level in Nanchang. The data were derived from the Nanchang land use map of 2014.

#### 2.2.2. Model Development and Evaluation 

In our study, twelve months were divided into: spring (March to May), summer (June to August), autumn (September to November), and winter (December to February). The LUR models of four seasons were developed, respectively, with SPSS Statistics 19.0 (IBM Corp., Armonk, NY, USA). The 24 samples of every season were randomly divided into two groups: a training data set and a test data set. A total of 75% of samples were used to develop the model and the remaining 25% were used for the model evaluation. The backward model-building algorithm proposed by Henderson et al. (2007) was introduced [35]. The steps were as follows: (1) correlation between PM_2.5_ and each independent variable was calculated through an individual univariate regression model; (2) variables that had a counter-intuitive correlation with PM_2.5_ were eliminated (e.g., traffic-related variables had negative coefficients and the ecological land proportion had a positive coefficient); (3) the highest-ranking variable in each subclass was identified and other subclass variables with a correlation of more than 0.6 with the highest-ranking variable were eliminated; (4) all remaining variables were entered into a stepwise linear regression; (5) the variables that had insignificant t-statistics (0.1) were removed (the t-statistics were lowered from 0.05 to 0.1 to control the meteorological variables); and (6) steps 4 and 5 were repeated until convergence was attained and variables that contributed less than 1% to the *R^2^* value of the final model were removed. The entire procedure was repeated three times for every season, and thus, three LUR models were developed for every season and the best fitting one was used as the final LUR model. In this way, the a priori division of samples could be avoided. The final LUR models were evaluated by comparing predicted PM_2.5_ concentrations with measured PM_2.5_ concentrations from the test data set. 

### 2.3. Selection of Urban Functional Zones

Five types of urban functional zones, including commercial, industrial, residential, educational, and control functional zones, were selected in the study area based on the Nanchang urban cadastral survey map and the Nanchang urban master planning map. When choosing urban functional zones, two rules were followed: (1) maintaining integral land parcels; (2) maintaining the evident land use.

In particular, the residential land accounted for more than 50% of the total residential functional zone area; the commercial land accounted for more than 60% of the total commercial functional zone area; and the industrial land (land for high-tech industry and storage included) accounted for more than 40% of the total industrial functional zone area. The land used for universities was chosen as an educational functional zone and one university was usually contained in an educational functional zone. Control functional zones included land use types, e.g., forest, water body, and farmland, and the area of these land use types accounted for more than 80% of the total control functional zone area.

### 2.4. Statistical Analysis 

Once the PM_2.5_ concentrations in the urban functional zones had been estimated, the analysis of variance and multiple comparisons test were carried out under the assumption of equal variances (homoscedasticity) and normal distribution. The statistical analysis was accomplished using SPSS Statistics 19.0. The analysis of variance can be used to test the null hypothesis *H*_0_, in which the PM_2.5_ concentrations in all functional zones have the same mean values, against the alternative hypothesis *H_1_*, where the mean values *μ_i_* of *k* groups are not the same. This can be written formally as follows [39].

(2) $$H_{0}:\mu_{1}=...=\mu_{k}=\mu \;H_{1}:not\;all\;the\;\mu_{i}\;are\;the\;same$$

The *F-ratio* and probability value (*p*-value) were obtained through a one-factor analysis of variance command. If *F > F (α*, *k* − *1*, *N* − *k)*, then *H*_1_ can be accepted. Additionally, a multiple comparison test is necessary to determine which group pairs’ mean values are significantly different. The least significant difference (LSD) test at a 0.05 level of probability was used to perform multiple comparisons. Using this method, the pairs of functional zones for which the PM_2.5_ concentrations are significantly different from each other can be identified.

## 3. Results

### 3.1. LUR Models

The final LUR models are reported in Table 3. Four variables were entered into the final LUR models after normalization, including meteorological factors, traffic-related factors, and land use factors. The variable of relative humidity was entered into the LUR models of spring, summer, and autumn (*p* < 0.01), and the variable of temperature was entered into the LUR models of spring and winter (*p* < 0.01). The intensity of the main roads within a 300 m buffer was found to be the dominant variable affecting PM_2.5_ pollution, because it was the only variable that entered all of the LUR models (*p* < 0.01). Land use factors including industrial land proportion and ecological land proportion also greatly impacted the PM_2.5_ concentrations, since they were entered into three LUR models (*p* < 0.1). The final models explained 76.4%, 89.9%, 94.1%, and 96.1% of the spatial variability of quarterly PM_2.5_ concentrations, respectively.

**Figure 2:**
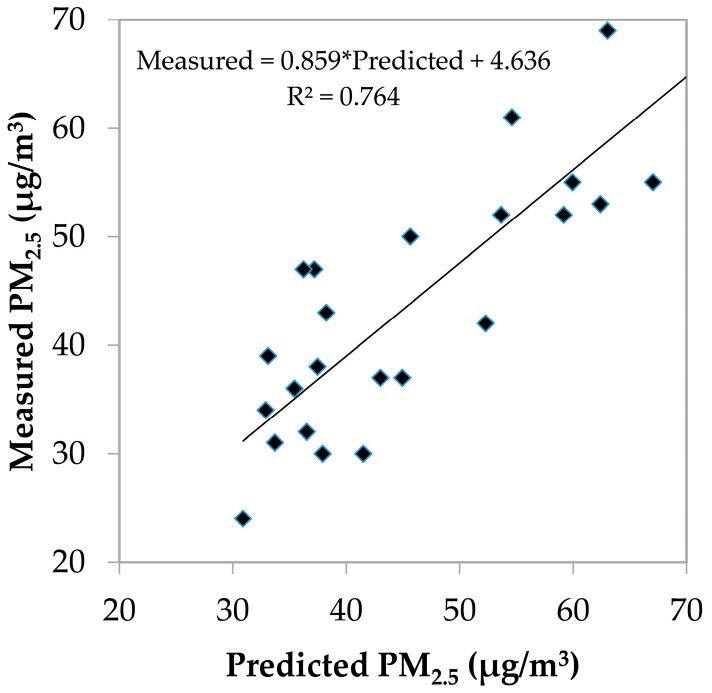
Predicted versus measured PM_2.5_ concentrations of the test data set.

To evaluate the performance of the final LUR models, the equations were applied to the test data set and the *R^2^* value between the predicted and measured PM_2.5_ concentrations was calculated. The *R^2^* value was 0.764. In addition, predicted data were plotted against measured data for validation (Figure 2). The figure shows that the predicted PM_2.5_ concentrations were well correlated with the measured concentrations. 

**Figure 3:**
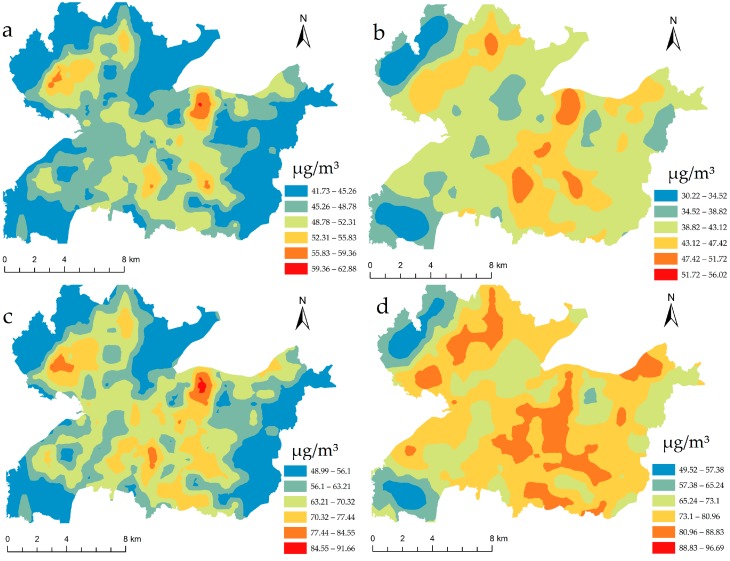
Final LUR models applied in the Nanchang urban area: PM_2.5_ concentrations in (**a**) Spring; (**b**) Summer; (**c**) Autumn; (**d**) Winter.

Grids with a dimension of 1 km × 1 km were created in the whole study area and the seasonal PM_2.5_ concentrations were calculated at each intersection using the final LUR models. We assumed there was no trend in the data and a spatially homogenous variation, and the seasonal spatial distributions of PM_2.5_ were then interpolated using the Ordinary Kriging approach. As shown in Figure 3, PM_2.5_ concentrations demonstrated a discernible spatial variation. High concentration areas occurred in the centre of the study area, while low concentration areas were mainly distributed on city borders. The northwest and southwest were low concentration areas throughout the year. The figure also discloses that the PM_2.5_ concentrations of most of the Nanchang urban area met the legislated 24-h average value, but exceeded the annual mean value, which are 75 μg/m^3^ and 35 μg/m^3^ in China, respectively.

### 3.2. Statistic Analysis of PM_2.5_ ConcentrationVariances among Different Types of Urban Functional Zones

**Figure 4:**
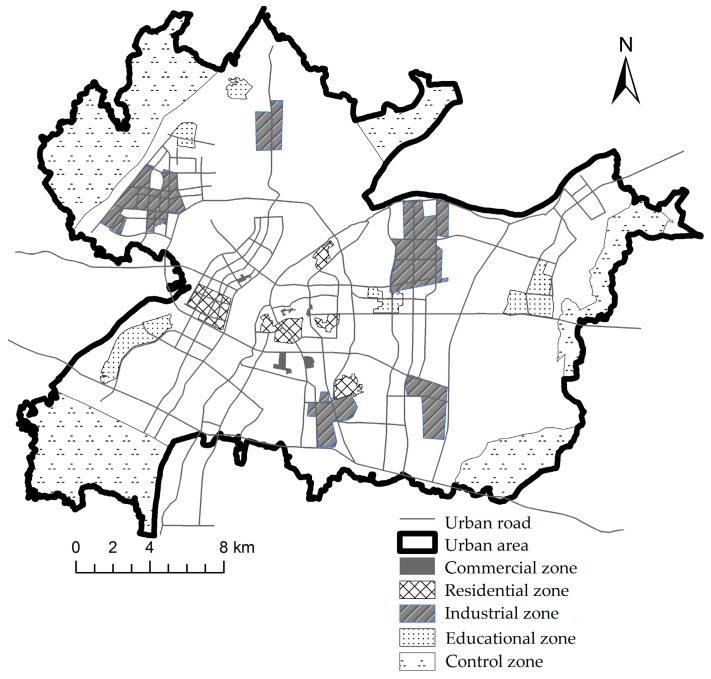
Location map of the urban functional zones in the Nanchang urban area.

Five types and a total of 25 urban functional zones were selected in the study area to analyze the PM_2.5_ concentration variances among different types of urban functional zones, as shown in Figure 4. The PM_2.5_ concentrations in the four seasons of these functional zones are shown in Table 4.

Analyses of the PM_2.5_ concentration variances in the urban functional zones were conducted after a normal distribution test and variance homogeneity test. Table 5 shows the one-factor variance analysis results. In spring, the *F-ratio* of 18.062 (*p* < 0.01) indicates that the PM_2.5_ concentration variances among different types of urban functional zones were significant. We can also conclude the same rule in summer, autumn, and winter.

Table 4 also expresses the multiple comparison results. In the four seasons, the multiple comparison results among different types of urban functional zones were the same. The results show that the PM_2.5_ concentration variances between the control and other four types of urban functional zones were significant. The PM_2.5_ concentration variances between the industrial or commercial functional zones and the residential or educational functional zones were also significant. However, there were no statistically significant PM_2.5_ concentration variances between the industrial and commercial functional zones, and the educational and residential functional zones. 

### 3.3. Statistic Analysis of PM_2.5_ Concentration Variances among the Same Type of Urban Functional Zones

Since residential land occupies the highest proportion of the urban area, the residential zone was selected as the typical functional zone to analyze PM_2.5_ concentration variances among the same type of urban functional zone. Another 15 residential functional zones were added to the original residential zone sample. Variables of intensity of the main roads, building volume rate, building density, and green coverage rate were used to build the multiple linear regression model for the annual PM_2.5_ prediction. As Table 6 shows, the model had a low fitting degree (adjusted *R*^2^ = 0.363). The intensity of the main roads positively correlated with PM_2.5_ concentrations and was the primary influencing variable in PM_2.5_ prediction (*p* < 0.01). The building volume rate was positively correlated with PM_2.5_ concentrations (*p* > 0.1) and the green coverage rate was negatively correlated with PM_2.5_ concentrations (*p* > 0.1). The building density showed a negative correlation with PM_2.5_ concentrations, which was counter-intuitive (*p* > 0.1). 

## 4. Discussion

### 4.1. LUR Models 

We developed LUR models incorporating meteorological factors for predicting quarterly PM_2.5_ concentrations in the Nanchang urban area, China. The adjusted *R^2^* values of the seasonal LUR models were 0.764, 0.899, 0.941, 0.961, respectively, explaining the spatial variability of the pollutant concentrations. In previous studies, the adjusted *R^2^* values of the LUR models ranged from 0.36 to 0.94 for PM_2.5_ [40,41]. The good performance of our models may be attributed to the combination of meteorological factors. Few LUR models include meteorological variables, although many studies have demonstrated that meteorology can significantly influence the pollutant concentration [23,24,25,26], possibly due to the lack of enough data or an appropriate methodology. Obtaining the meteorological conditions at each monitoring site is costly and time-consuming. In this study, we presupposed an identical meteorological condition at every site, as the study area was not very large. Different meteorological factors were entered into the LUR models of different seasons, demonstrating that the influence of meteorological factors on PM_2.5_ concentrations varied as the seasons changed. 

Among all the independent variables, the intensity of main roads within a 300 m buffer was the dominant variable affecting PM_2.5_ concentrations, indicating that PM_2.5_ concentrations are closely related to traffic conditions. Some studies used vehicle intensity, while other studies used road length or road intensity to represent traffic conditions [26,42,43,44,45]. Compared to road length or road intensity, vehicle intensity is more representative of vehicle exhaust, but the data are often unavailable for researchers because of the high cost of vehicle monitoring. Studies have also proved that the performance of LUR models developed with road length or road intensity didn’t differ from those developed with vehicle intensity [35,46]. Therefore, road intensity was used in our models in the absence of vehicle intensity. The independent variable of industrial land proportion increasing PM_2.5_ pollution in other studies was also included in the LUR models [33,42]. The variables of road intensity and industrial land proportion implying sources of PM_2.5_ in Nanchang are mainly local transportation and major industries. The independent variable of ecological land proportion decreasing PM_2.5_ pollution in other Chinese cities was also included in the LUR models [33,47], suggesting that the function of natural spaces in removing pollutants is evident. It should be noticed that the independent variable of population density increasing PM_2.5_ concentration in other Chinese cities was not included in our models [33,47]. The reason for this is that the spatial resolution of the variable was not good enough in our study.

The number of monitoring sites might be an important factor influencing the accuracy of LUR models. However, at present, there is no rigorous methodology to determine the number of required monitoring sites. The population and size of cities are generally thought to be taken into account when determining the actual number of monitoring sites [40]. In our study, there were eight monitoring sites and the coverage area was 562.46 km^2^, resulting in a monitoring density of one site for every 70 km^2^. Although it was a small number of monitoring sites, the spatial coverage was comparable to other LUR models reported in the literature [33,35,37,42,46].

### 4.2. Impact of Land Use on PM_2.5_ Pollution

The paper studied the impact of land use on PM_2.5_ pollution from two aspects of land use type and land use intensity. The impact of land use type on PM_2.5_ pollution was investigated by analyzing the PM_2.5_ concentration variances among different types of urban functional zones. Through the analysis of PM_2.5_ data from different types of functional zones, the same rule in four seasons was found. The highest PM_2.5_ concentration was found in industrial and commercial functional zones, while the lowest occurred in control functional zones. The PM_2.5_ concentration in residential and educational functional zones was in between these zone types. PM_2.5_ pollution in the commercial zone was relatively high in comparison with industrial functional zones, and the residential zone was slightly higher than educational functional zones. The PM_2.5_ concentrations in different types of functional zones have also been investigated through a sample survey and a similar pattern has been found [48], which confirms the high simulation accuracy of the final LUR models. Further, our results demonstrate that the PM_2.5_ concentration variances among different urban functional zones were statistically significant. The significant PM_2.5_ variances suggest that the PM_2.5_ pollutants in the Nanchang urban area mainly come from local transportation and major industries, echoing the results demonstrated in the LUR models. We can also conclude that the urban functional zones which are characterized by a dominant land use type had a great impact on PM_2.5_ pollution and the impact did not change as the seasons changed. 

The impact of land use intensity on PM_2.5_ pollution was investigated through predicting annual PM_2.5_ concentrations with indexes including the building volume rate, building density, and green coverage rate. The concept of land use intensity is far from an innovative term and first appeared in David Ricardo’s Land Rent Theory, which is similar to concepts of smart growth, compact city, Infill Development, and Urban Growth Boundary [49]. In China, land use intensity is considered as the national guideline to alleviate the demand for urban land driven by economic and population growth. However, studies have shown that a higher land use intensity leads to more prominent environmental problems, like noise, dust, and toxic pollutants, because a higher land use intensity increases the concentration of the urban activities [50]. In our paper, the multiple linear regression results showed insignificant t-statistics and inconsistent coefficients with a priori assumptions, illustrating that the indexes had an insignificant or counter-intuitive impact on PM_2.5_ concentrations. This may due to the complex physical-chemical mechanism of PM_2.5_ pollution or the improper study spatial scale.

In our paper, urban functional zones were used as the basic research unit to explore the effect of land use on PM_2.5_ pollution. Some studies analyzed the effect of land use on PM_2.5_ pollution through calculating the correlation between PM_2.5_ pollution and land use/land cover types [34,51,52]. Compared to a single land use type, urban functional zones including a variety of land use types, but characterized by a dominant land use type, are more appropriate for urban areas. Scholars also believe that urban functional zones can better reflect the relationship between urban land use and air pollution as its specific social-economic function [30]. The results demonstrate that the urban functional zone was a proper spatial scale to investigate the impact of land use type on PM_2.5_ pollution in urban areas. 

### 4.3. Limitations

There are some limitations that need to be addressed. The first limitation of this study was the weakness related to applying the LUR model to a large area. As the study area in our paper is not very large, we presupposed identical meteorological conditions at every monitoring site. In large areas, the meteorological variables vary from one monitoring site to another and will show a differential influence at each site. Secondly, only a one-year period was considered in this paper due to the data access limitations. Using data from longer periods can help improve the prediction ability of an LUR model. Lastly, it should be noted that this research has explored the impact of land use on PM_2.5_ pollution through analyzing the intra-urban spatial variability of PM_2.5_ concentrations. Further research is needed to investigate the detailed mechanisms of how land use influences PM_2.5_ concentration.

## 5. Conclusions

This paper attempted to use LUR models to simulate the variances of the PM_2.5_ level in the Nanchang urban area and statistical analysis to explore the impact of land use on PM_2.5_ pollution. The seasonal LUR models showed a good fit and could explain the spatial variability in PM_2.5_ concentrations well. PM_2.5_ exhibits a large variation in different seasons, with the highest pollution values in winter and the lowest in summer, due to the complicated influence of the meteorological factors of temperature and relative humidity [53,54]. Similar to many other studies, the dominant PM_2.5_ impacting variable was the traffic conditions that were characterized by the road intensity in this paper [37,42,55,56,57]. The analysis of variance and multiple comparison test shows statistically significant variances in PM_2.5_ concentrations among different types of urban functional zones throughout the year, demonstrating that the land use types generated a great impact on PM_2.5_ concentrations and the impact did not change as the seasons changed. The multiple linear regression results illustrate that the land use intensity indexes including the building volume rate, building density, and green coverage rate exhibited an insignificant or counter-intuitive impact on PM_2.5_ concentrations. The study also concludes that the urban functional zone was a proper spatial scale to investigate the impact of land use type on PM_2.5_ pollution in urban areas, but might not be a proper spatial scale to explore the impact of land use intensity on PM_2.5_ pollution. A reasonable methodology and optimized spatial scale are still yet to be explored to further investigate how land use intensity affects PM_2.5_ pollution.

## Figures and Tables

**Table 1:** The time-serial fine particulate matter (PM_2.5_) concentrations for the eight monitoring sites in 2014.

Monitoring Site	Month (μg/m^3^)											
	Jan.	Feb.	Mar.	Apr.	May	Jun.	Jul.	Aug.	Sep.	Oct.	Nov.	Dec.
S1	105	38	46	45	61	50	36	34	52	92	74	61
S2	109	38	44	37	45	39	27	25	36	67	54	52
S3	119	41	45	48	71	55	38	38	57	92	62	55
S4	102	37	44	47	69	55	36	37	52	92	61	54
S5	88	27	36	30	42	39	27	24	35	58	38	38
S6	97	32	36	37	60	47	33	31	44	81	55	51
S7	96	39	47	40	48	46	42	34	38	53	50	36
S8	87	30	37	39	49	55	47	43	59	81	60	57

**Table 2:** The description of independent variables.

Variable	Unit	Max	Min	Mean	SD
Relative humidity	%	80	57	74.167	8.077
Air pressure	hpa	1022.3	998.8	1009.750	7.994
Water vapor pressure	hpa	31.8	5.8	18.075	9.167
Temperature	°C	29	7.3	18.817	8.183
Wind speed	m/s	1.9	1.4	1.675	0.166
Intensity of main roads (300–4800 m)	m/m^2^	0.270	0	0.113	0.061
Intensity of secondary roads (300–4800 m)	m/m^2^	0.428	0	0.085	0.100
Intensity of total roads (300–4800 m)	m/m^2^	0.644	0	0.220	0.115
Ecological land proportion (300–4800 m)	%	99.728	5.483	39.027	20.233
Industrial land proportion (300–4800 m)	%	53.515	0	11.433	14.699
Distance to large ecological space	m	1826	67	827.500	659.948
Residential land proportion (300–4800 m)	%	49.015	0	18.438	11.973

Note: SD means standard deviation.

**Table 3:** The final land use regression (LUR) models for PM_2.5_ concentrations in the Nanchang urban area.

Season	Model Variable	*β*	*SE*	*p*	*VIF*	*Adj. R*^2^	*SE*
Spring	Intercept	46.722	1.050	0.000		0.764	4.455
	Intensity of main roads (300 m)	4.859	1.085	0.001	1.009		
	Industrial land proportion (300 m)	2.087	1.110	0.083	1.055		
	Temperature	16.748	3.061	0.000	8.023		
	Relative Humidity	11.521	3.049	0.002	7.963		
Summer	Intercept	40.056	0.718	0.000		0.899	3.044
	Intensity of main roads (300 m)	3.008	0.763	0.002	1.067		
	Industrial land proportion (300 m)	3.103	0.858	0.054	1.351		
	Ecological land proportion (2400 m)	−3.159	0.925	0.058	1.570		
	Relative Humidity	−6.846	0.775	0.000	1.102		
Autumn	Intercept	62.000	1.044	0.000		0.941	4.429
	Intensity of main roads (300 m)	9.469	1.149	0.000	1.143		
	Industrial land proportion (300 m)	3.213	1.147	0.056	1.141		
	Ecological land proportion (300 m)	−2.825	1.199	0.058	1.247		
	Relative Humidity	−14.150	1.156	0.000	1.159		
Winter	Intercept	67.778	1.461	0.000		0.961	6.198
	Intensity of main roads (300 m)	4.805	1.541	0.008	1.051		
	Distance to large ecological space	3.380	1.690	0.067	1.264		
	Ecological land proportion (2400 m)	−4.362	1.723	0.020	1.313		
	Temperature	30.048	1.513	0.000	1.014		

Note: *β* is the associated coefficient of the LUR model, VIF means variance inflation factors.

**Table 4:** PM_2.5_ concentrations in the functional zones in four seasons.

Functional Zone Type	Mean ± SD/(μg·m^−3^)			
	Spring	Summer	Autumn	Winter
Commercial zones	53.396 ± 1.23a	44.476 ± 0.89a	75.062 ± 2.48a	82.702 ± 1.37a
Industrial zones	51.734 ± 1.11a	43.272 ± 0.81a	71.724 ± 2.23a	80.856 ± 1.23a
Educational zones	46.674 ± 0.95b	39.806 ± 0.64b	61.402 ± 1.98b	65.498 ± 3.23b
Residential zones	47.914 ± 1.16b	40.502 ± 0.84b	64.056 ± 2.32b	68.624 ± 1.28b
Control zones	42.500 ± 0.37c	36.574 ± 0.27c	53.174 ± 0.74c	58.842 ± 0.34c

Note: Different lowercase letters in the same column indicate significantly different PM_2.5_ concentrations in functional zones of the same season at 5%.

**Table 5:** Variance analysis results.

Season	Variable	Squares Sum	Freedom	Mean Square	*F-Ratio*	*p*-Value
Spring	Between-group	370.695	4	92.674	18.062	0
	Within-group	102.616	20	5.131		
	Total	473.31	24			
Summer	Between-group	192.401	4	48.1	18.264	0
	Within-group	52.673	20	2.634		
	Total	245.074	24			
Autumn	Between-group	1500.56	4	375.14	17.888	0
	Within-group	419.443	20	20.972		
	Total	1920.004	24			
Winter	Between-group	313.688	4	78.422	18.259	0
	Within-group	85.901	20	4.295		
	Total	399.589	24			

**Table 6:** Multiple linear regression model for annual PM_2.5_ concentrations in residential zones.

Model Variable	*β*	*SE*	*p*-Value	*VIF*	*Adj. R*^2^	*SE*
Intercept	52.178	4.059	0		0.363	4.707
Intensity of main roads (300 m)	56.197	16.236	0.003	1.425		
Building volume rate	0.577	0.996	0.555	2.308		
Building density	−0.016	0.059	0.785	1.991		
Green coverage rate	−0.181	0.162	0.281	1.636		
